# Ulinastatin Protects Cognition in Cardiac Surgical Patients

**DOI:** 10.5761/atcs.oa.26-00135

**Published:** 2026-10-14

**Authors:** Yan Hu, Yile Zhou, Huihui Ju, Xianfu Lu, Yi Shi, Hao Fang

**Affiliations:** 1 Department of Anesthesiology, Zhongshan Hospital, Fudan University, Shanghai, China; 2 Department of Anesthesiology, Zhongshan Hospital (Xiamen), Fudan University, Xiamen, China; 3 Department of Anesthesiology (High-Tech Branch), The First Affiliated Hospital of Anhui Medical University, Hefei, China; 4 Department of Kidney Transplantation, Zhongshan Hospital, Fudan University, Shanghai, China; 5 Shanghai Key Laboratory of Organ Transplantation, Zhongshan Hospital, Fudan University, Shanghai, China

**Keywords:** ulinastatin, cardiac surgery, cognitive dysfunction, inflammation

## Abstract

**Purpose:**

This study aimed to investigate the incidence of postoperative cognitive dysfunction (POCD) in patients undergoing cardiac valve surgery and the potential protective effects of ulinastatin on POCD.

**Methods:**

This was a prospective, multicenter, randomized, double-blind clinical trial. A total of 300 patients undergoing cardiac valve surgery were enrolled across the 3 hospitals and randomized to the control or ulinastatin treatment groups. The patients’ cognition was evaluated using the Mini-Mental State Examination (MMSE). POCD was defined as a lower cognitive score after surgery than the baseline score. Serum levels of proinflammatory cytokines were measured by an enzyme-linked immunosorbent assay.

**Results:**

The incidence of POCD in the control group was significantly higher than those in the ulinastatin group (42/150 vs. 24/150, p <0.05). The MMSE score in the control group was significantly lower after the operation [D0 vs. D3: 27.72 (1.88) vs. 27.09 (2.61), p <0.05], but did not change in the ulinastatin group [D0 vs. D3: 27.63 (2.29) vs. 27.31 (2.47), p >0.05]. In the ulinastatin group, plasma levels of matrix metallopeptidase 9 (MMP9) and S100 calcium-binding protein B (S100β) were significantly lower than those in the control group after the operation.

**Conclusions:**

The present study confirms that a transient proinflammatory state impairs cognition in patients undergoing cardiac surgery and indicates that perioperative administration of ulinastatin protects cognition.

## Introduction

Postoperative cognitive dysfunction (POCD) refers to cognitive decline after surgery, manifested as impaired memory and attention, abnormal information processing, and executive dysfunction.^1)^ Approximately 50% of patients are diagnosed with cognitive dysfunction after cardiac surgery, which is higher among elderly patients.^2)^ The underlying mechanisms of POCD involve many pathological factors; therefore, POCD is diagnosed by the exclusion of neurological diseases, such as dementia and stroke, and anesthetic medicine-induced residual effects. Aging is an independent risk factor for the occurrence of the disorder, probably due to moderate neuronal loss, vascular dysfunction, mildly elevated levels of inflammation, and increased sensitivity to narcotic drugs during the process of aging. Other pathological conditions include an increased release of inflammatory factors, tissue hypoperfusion, and microthrombus formation.^3,4)^

With aging, the central nervous system is compromised by chronic hypoxia and hypoperfusion, neuronal dysfunction and loss, astrocyte activation, and blood–brain barrier impairment.^5)^ The combined challenges of surgery and anesthesia further increase serum levels of inflammatory factors and worsen cognitive impairment in elderly humans and aged animal models.^6)^ Elevated pro-inflammatory immune profiles with impaired cognitive performance have been reported in elderly patients. Patients with POCD have increased levels of interleukin-1 beta (IL-1β), interleukin-6 (IL-6), and tumor necrosis factor-alpha (TNFα) in the circulation and the central nervous system, indicating that systemic pro-inflammatory cytokines probably disrupt the blood–brain barrier and stimulate inflammation in the central nervous system, thereby creating a vicious cycle.^7)^ Thus, early intervention in perioperative inflammation can reduce the occurrence of POCD.^8,9)^

Ulinastatin, which is extracted from human urine, exerts anti-inflammatory effects by stabilizing lysosomal membranes.^10)^ Ulinastatin has been applied in the treatment of pancreatic and cranial diseases. It has also been reported that ulinastatin treatment improves cognitive impairment in rodent models of global cerebral ischemia–reperfusion injury by reducing the neuroinflammatory response.^10,11)^

The Mini-Mental State Examination (MMSE) is commonly used in clinical practice. By examining orientation, registration, attention and calculation, recall, and language, the MMSE is an easy and critical tool to check mental skills and track changes over time.^12)^

Since the construction of core cognition lacks consensus, subjective judgment impacts examination results.^13)^ Thus, hand in hand with the MMSE evaluation, several potential inflammatory cytokines and chemokines were also examined in the present study.

The present study was designed to investigate the involvement of inflammatory factors in POCD in a prospective, randomized, double-blind clinical study.^14)^ It was hypothesized that ulinastatin, a clinical intervention, inhibits inflammation in patients undergoing cardiac surgery and protects against cognitive impairment.

## Materials and Methods

### Patients and design

The present study was registered as a prospective, multicenter, randomized, double-blind clinical trial. The Zhongshan Hospital Ethics Committee approved the study protocol (B2021-016R). Written informed consent was obtained from patients before surgery. A copy of the written consent form is available for review by the editor-in-chief of this journal upon request. A total of 330 patients undergoing cardiopulmonary bypass in the 3 hospitals were enrolled from July 2021 to March 2023.

### Patients and public involvement

The present project was designed and conducted by medical staff. There was no patient or public involvement in the design, conduct, reporting, or dissemination.

### Inclusion and exclusion criteria

Patients in the present study who had valve diseases, including aortic stenosis/regurgitation and mitral stenosis/regurgitation, were scheduled for valve surgery under cardiopulmonary bypass. The recruitment criteria included being 45–75 years old, having completed primary education, and having an ASA evaluation of III–IV. Exclusion criteria included both emergency and chronic clinical conditions. Acute exclusion conditions were progressive heart failure, stroke, myocardial infarction, and traffic accident, as well as systemic infections requiring antibiotic treatment within 1 week. Chronic medical conditions, such as body mass index greater than 30 kg/m^2^, uncontrolled diabetes and/or hypertension, neurological diseases, and any other inappropriate disease conditions as determined by the researchers, were also excluded from the present study.

### Data collection and preparation

Data were collected using investigator-initiated trial electronic data capture (Zhejiang Taimei Medical Technology, Jiaxing, China). The sample size was calculated using G* Power software, with a 2-sided significance level of 5% (α = 0.05) and 80% power. All participants completed the MMSE before surgery (D0), 1 day after surgery (D1), and 3 days after surgery (D3). Patients were randomized into the control group (Group C) and the ulinastatin group (Group U). The randomization sequence without stratification was generated by a computer and sealed in consecutively numbered envelopes. An independent study nurse was responsible for preparing the ulinastatin. Patients, statisticians, and medical staff, including cardiac surgeons, anesthetists, and critical care staff, were blinded to the assignment until completion of the final analysis.

### Outcomes

The primary outcome was a decline in cognition, defined in the present study as a decrease in the MMSE score of more than 2 points from baseline.^15)^ The secondary outcome was the incidence of adverse events after surgery, including abnormal laboratory parameters, mechanical ventilation support, and complications.

### Anesthesia

All patients received alprazolam and ranitidine 30 minutes before surgery. Two catheters were placed in the right internal jugular vein and radial artery. Anesthesia was induced with a combination of propofol (2 μg/ml), sufentanil (0.5 μg/kg), and rocuronium (1 mg/kg), and was maintained with continuous propofol infusion and sevoflurane inhalation. The anesthesia status was monitored using an electroencephalogram (bispectral index of 40–60) and by maintaining a mean arterial pressure >60 mmHg during the operation. Transesophageal echocardiography was used for intraoperative cardiac assessment. The minimum temperature during cardiopulmonary bypass was about 33°C–36°C. Del Nido cardioplegia was used for circulatory arrest.

Patients received their first dose of ulinastatin (10000 units/kg/day) via intravenous infusion before cardiopulmonary bypass on the day of surgery (D0) and then received ulinastatin infusion for 7 days after surgery or until discharge. The control group received the same amount of saline.

### Laboratory examination

Serum samples were obtained before surgery (D0), 1 day after surgery (D1), and 3 days after surgery (D3). Serum samples were centrifuged at 2500 rpm for 15 min and stored at −80°C for further measurement. Serum levels of C-reactive protein (CRP), matrix metallopeptidase 9 (MMP9), S100 calcium-binding protein B (S100β), interferon gamma (IFNγ), TNFα, and C–X–C motif chemokine ligand 10 (CXCL10, also known as IP-10) were measured using enzyme-linked immunosorbent assay kits (ELISA, R&D Systems, Brea, CA, United States).

### Statistical analysis

Data are presented as mean (standard deviation, SD). Statistical analyses were performed by SPSS 26.0. The statistical description included the number of subjects, the means, and the standard deviations of the measurement data. A stratified chi-square test (CMH χ^2^) was used to compare incidences between the ulinastatin and control groups (α = 0.05). P <0.05 was considered statistically significant.

## Results

### Baseline of clinical data

The present study recruited 330 patients from July 2021 to March 2023. Fifteen patients were excluded before surgery, and 15 patients were withdrawn during the study (**Fig. 1**). A total of 300 patients were randomized into the control and ulinastatin groups (control vs. ulinastatin: 150 vs. 150; **Fig. 1**). General condition and clinical characteristics were comparable in the 2 groups (**Table 1**).

**Fig. 1:**
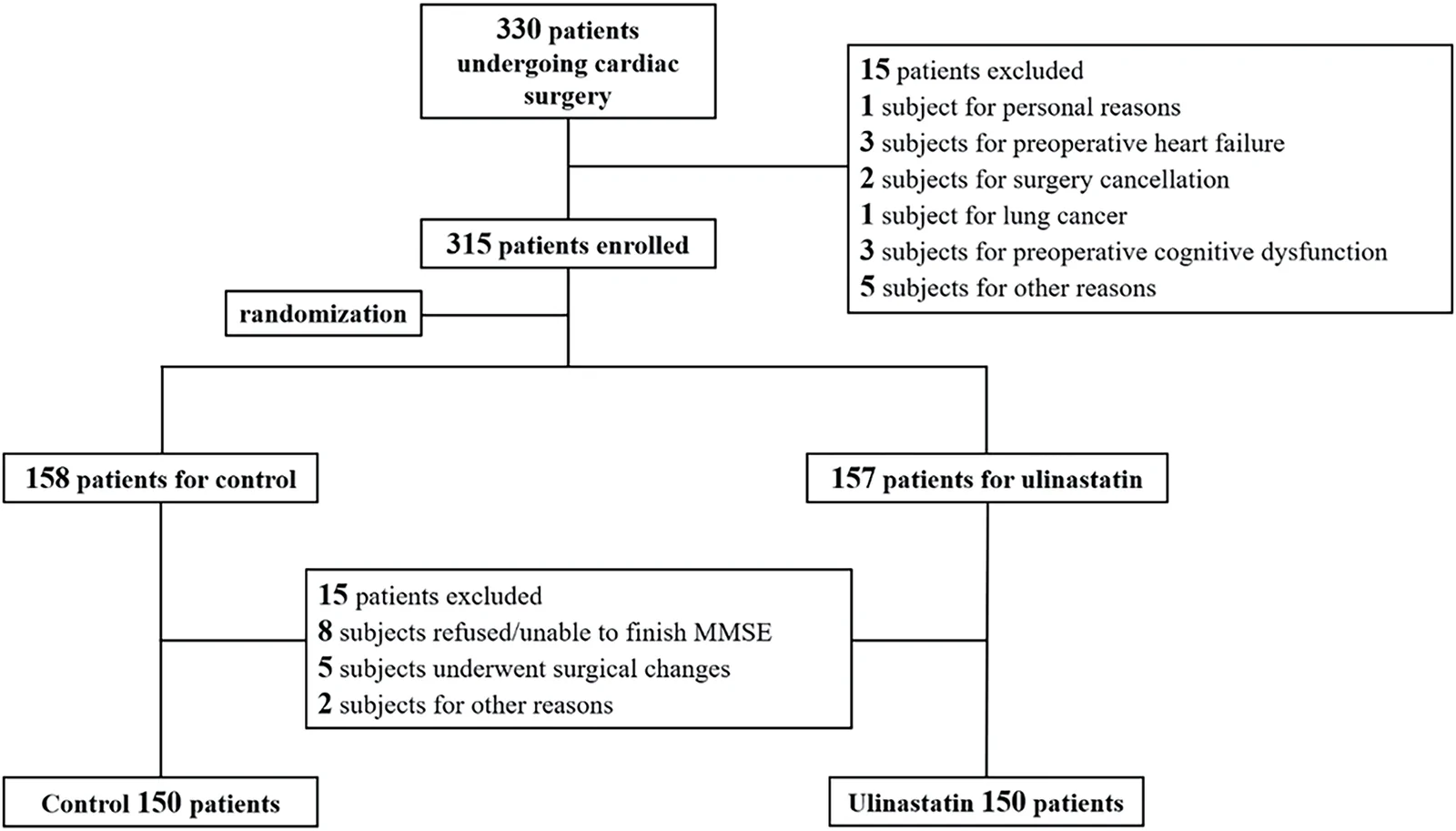
Screening and randomization flowchart for the present study.

**Table 1:** General conditions and clinical characteristics of patients

	Control (n = 150)	Ulinastatin (n = 150)	P-value
Age (year)	59.29 (7.11)	59.43 (7.54)	0.87
BMI (kg/m^2^)	23.42 (2.92)	24.08 (3.45)	0.08
Gender (F/M)	57/93	55/95	0.91
Hypertension and/or diabetes	30/120	21/129	0.22
Preoperative MMSE	27.72 (1.88)	27.63 (2.29)	0.71
Laboratory examination			
RBC	4.429 (0.58)	4.331 (0.61)	0.16
Hb	133.5 (22.58)	133.2 (14.91)	0.91
WBC	6.08 (1.57)	5.89 (1.70)	0.31
PLT	190.7 (61.45)	181.5 (63.21)	0.21
ALT	23.12 (15.79)	22.99 (20.37)	0.95
AST	23.14 (11.33)	22.74 (10.93)	0.76
BUN	7.71 (9.60)	7.42 (6.86)	0.77
Cr	75.88 (16.66)	79.54 (51.12)	0.42
Preoperative ECHO			
EF (%)	60.84 (7.96)	61.72 (7.06)	0.31
PAP (mmHg)	43.18 (12.15)	40.99 (12.26)	0.13
Intraoperative conditions			
Anesthesia duration (min)	324.9 (87.28)	314.2 (95.18)	0.31
Surgical duration (min)	254 (84.58)	245.4 (85.19)	0.67
Aortic occlusion duration (min)	85.9 (37.29)	82.03 (36.41)	0.37
CPB duration (min)	127.9 (48.87)	121.1 (44.11)	0.21
Crystalloid solution volume (ml)	1326 (416.2)	1283 (477.4)	0.41
Colloidal solution volume (ml)	483.7 (116)	479.2 (143.6)	0.81
Urine volume (ml)	744.3 (711.7)	747.6 (684.4)	0.97
Blood transfusion			
RBC (U), (n)	2.74 (1.02) (n = 21)	2.35 (0.93) (n = 17)	0.24
Plasma (ml), (n)	467.9 (176.4) (n = 24)	491.7 (252.8) (n = 18)	0.72

Data are presented as mean (standard deviation, SD).

BMI, body mass index; MMSE, mini-mental state examination; RBC, red blood cell; Hb, hemoglobin; WBC, white blood cell; PLT, platelet; ALT, alanine aminotransferase; AST, aspartate aminotransferase; BUN, blood urea nitrogen; Cr, creatinine; ECHO, echocardiography; EF, ejection fraction; PAP, pulmonary artery pressure; CPB, cardiopulmonary bypass

### Primary outcome

Before surgery, the MMSE score was 27.72 (1.88) in the control group and 27.63 (2.29) in the ulinastatin group (p = 0.71). On Day 3, the MMSE score in the control group decreased [D0 vs. D3: 27.72 (1.88) vs. 27.09 (2.61), p <0.05], while the MMSE scores in the ulinastatin group did not change significantly [D0 vs. D3: 27.63 (2.29) vs. 27.31 (2.47), p = 0.25]. A total of 42 patients in the control group and 24 patients in the ulinastatin group were diagnosed with POCD (control vs. ulinastatin: 42/150 vs. 24/150, p <0.05; **Table 2** and **Fig. 2**).

**Table 2:** Comparison of the MMSE scores in the control and ulinastatin-treated groups

	Control (n = 150)	Ulinastatin (n = 150)	P-value C vs. U
Preoperative MMSE	27.72 (1.88)	27.63 (2.29)	0.71
Postoperative MMSE	27.09 (2.61)	27.31 (2.47)	0.47
P-value D3 vs. D0	0.02*	0.25	

*P <0.05 compared with the baseline of the same group.

Data are presented as mean (standard deviation, SD).

Data are presented as mean ± standard deviation.

MMSE, mini-mental state examination

**Fig. 2:**
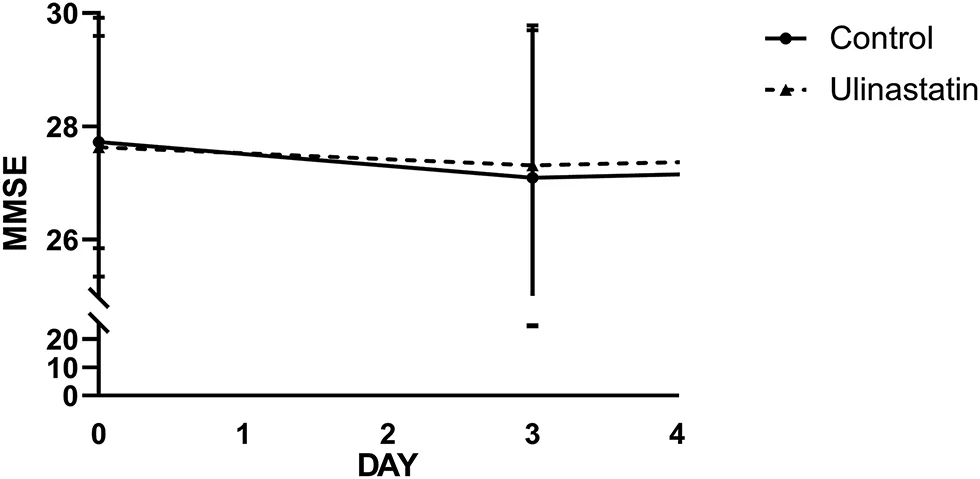
Changes in MMSE scores during the perioperative period. Data are presented as mean ± standard deviation.

### Secondary outcome

The monitoring parameters during surgery were comparable, including heart rebeating (control vs. ulinastatin: 86% vs. 88%) and mechanical ventilation time [control vs. ulinastatin: 980.1 (667.8) min vs. 976.8 (621.7) min] (**Table 3**).

**Table 3:** Surgery and anesthesia-associated parameters in patients

	Control (n = 150)	Ulinastatin (n = 150)	P-value
Cardiac re-beating (defibrillation/automatic)	21/129	18/132	0.73
Postoperative ECHO			
EF (%)	56.51 (8.01)	58 (7.58)	0.18
PAP (mmHg)	33.97 (6.68)	34.46 (8.46)	0.67
Laboratory examination			
RBC	3.35 (0.61)	3.35 (0.68)	0.98
Hb	102.2 (19.98)	101.4 (16.49)	0.73
WBC	12.23 (7.31)	11.78 (4.35)	0.54
PLT	117.4 (43.94)	116.4 (44.34)	0.86
ALT	27.9 (27.12)	31.73 (61.64)	0.52
AST	47.01 (46.53)	45.25 (54.75)	0.78
BUN	8.01 (3.62)	9.85 (19.46)	0.29
Cr	74.69 (32.16)	73.99 (30.36)	0.86
Postoperative conditions			
Mechanical ventilation time (min)	980.1 (667.8)	976.8 (621.7)	0.97
ICU duration (min)	1934 (1470)	1927 (1917)	0.97
Hospital stay (day)	15.47 (7.57)	17.17 (10.50)	0.11

Data are presented as mean (SD).

ECHO, echocardiography; EF, ejection fraction; PAP, pulmonary artery pressure; RBC, red blood cell; Hb, hemoglobin; WBC, white blood cell; PLT, platelet; ALT, alanine aminotransferase; AST, aspartate aminotransferase; BUN, blood urea nitrogen; Cr, creatinine; ICU, intensive care unit

After surgery, both groups had lower red blood cell and platelet counts, higher white blood cell counts, and increased serum aspartate aminotransferase levels, which were comparable in the 2 groups (**Table 4**).

**Table 4:** Changes in laboratory examination before and after surgery

	Preoperative			Postoperative			P-value (control)	P-value (Ulinastatin)
	Control	Ulinastatin	P-value	Control	Ulinastatin	P-value	Pre vs. post	Pre vs. post
Laboratory examination								
RBC	4.429 (0.58)	4.331 (0.61)	0.16	3.35 (0.62)	3.351 (0.68)	0.98	<0.0001	<0.0001
Hb	133.5 (22.58)	133.2 (14.91)	0.90	102.2 (19.98)	101.4 (16.49)	0.72	<0.0001	<0.0001
WBC	6.084 (1.57)	5.888 (1.70)	0.31	12.23 (7.31)	11.78 (4.35)	0.54	<0.0001	<0.0001
PLT	190.7 (61.45)	181.5 (63.21)	0.21	117.4 (43.94)	116.4 (44.34)	0.86	<0.0001	<0.0001
ALT	23.12 (15.79)	22.99 (20.37)	0.95	27.9 (27.12)	31.73 (61.64)	0.51	0.07	0.11
AST	23.14 (11.33)	22.74 (10.93)	0.76	47.01 (46.53)	45.25 (54.75)	0.78	<0.0001	<0.0001
BUN	7.71 (9.60)	7.42 (6.86)	0.77	8.01 (3.62)	9.85 (19.46)	0.29	0.74	0.17
Cr	75.88 (16.66)	79.54 (51.1)	0.41	74.69 (32.16)	73.99 (30.36)	0.86	0.70	0.29
cTnT	0.01 (0.01)	0.01 (0.02)	0.42	0.42 (0.46)	0.45 (0.44)	0.78	<0.0001	<0.0001
CK-MB	9.6 (7.55)	9.99 (9.59)	0.80	11.66 (8.58)	24.07 (54.44)	0.09	0.17	0.05
Pro-BNP	533.7 (722.0)	570.8 (737.8)	0.75	1646 (1004)	1908 (3189)	0.56	<0.0001	0.0006

Data presented as mean (SD).

RBC, red blood cell; Hb, hemoglobin; WBC, white blood cell; PLT, platelet; ALT, alanine aminotransferase; AST, aspartate aminotransferase; BUN, blood urea nitrogen; Cr, creatinine

In the present study, none of the patients experienced severe surgical complications or systemic infections (**Table 3**).

### Measurement of inflammatory factors

Six serum inflammatory factors were measured. Before surgery, the serum levels of CRP, MMP9, S100β, IFNγ, TNFα, and IP-10 were comparable in the 2 groups. Compared with the baseline measurements, the control group had transiently increased levels of CRP and IFNγ, transiently reduced levels of MMP9, and comparable levels of TNFα and IP-10. In the ulinastatin group, the serum MMP9 level, but not other circulating factors, was slightly reduced on D1. On D3, MMP9 and S100β levels in the ulinastatin group were slightly reduced compared to those in the control group (**Fig. 3**).

**Fig. 3:**
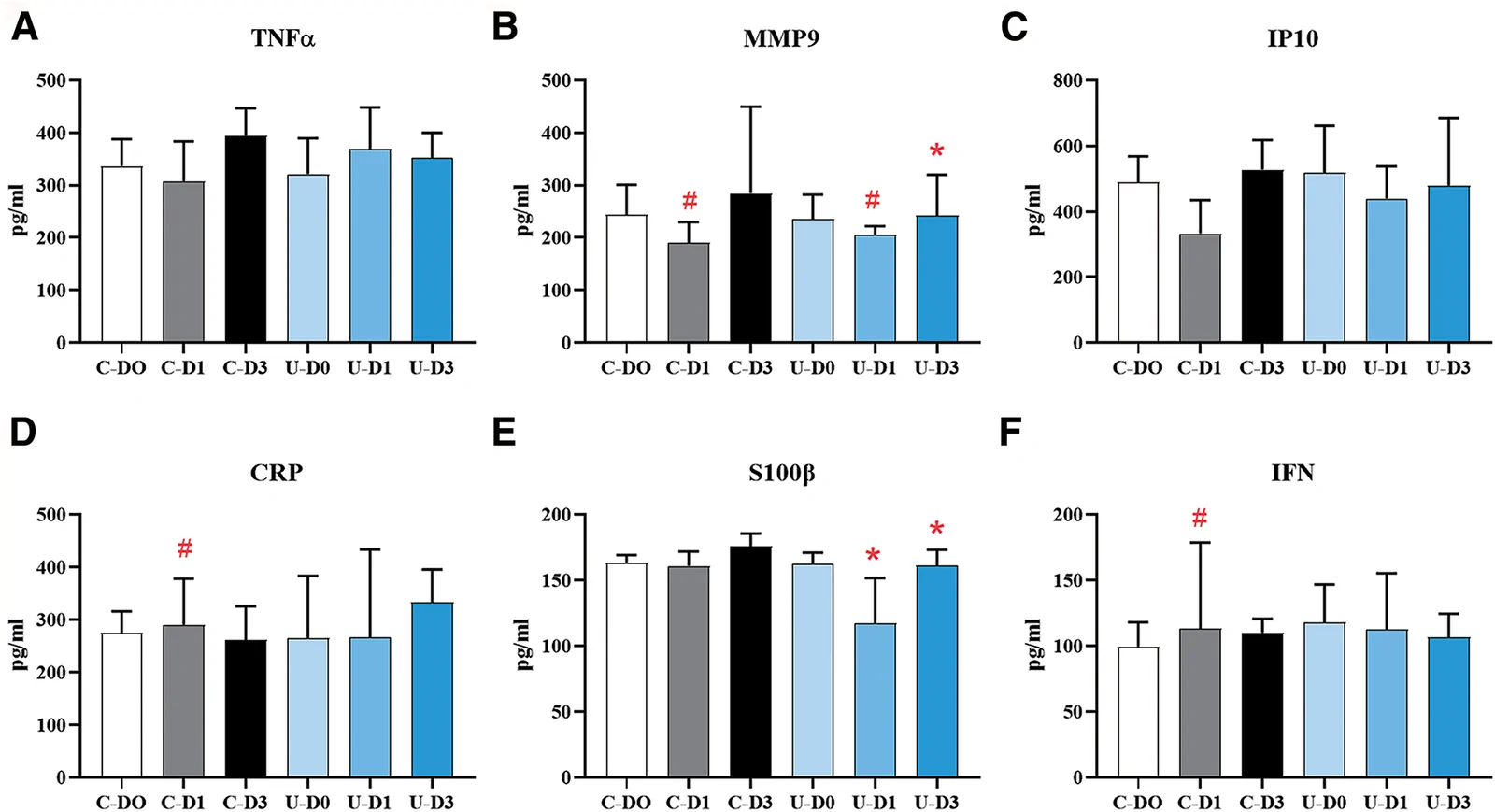
Changes in inflammatory factors during the perioperative period. **A**, TNFα in serum during the perioperative period; **B**, MMP9 in serum during the perioperative period; **C**, IP10 in serum during the perioperative period; **D**, CRP in serum during the perioperative period; E, S100β, in serum during the perioperative period; F, IFNγ, in serum during the perioperative period. Data are shown as mean ± standard deviation. ^#^P <0.05 compared with the baseline value of the same group. *P <0.05 compared with the control group under the same condition.

## Discussion

The present study reports that about 22% of patients undergoing cardiac surgery exhibit cognitive decline. Medical intervention with ulinastatin moderately mitigates cognitive impairment by reducing serum levels of MMP9 and S100β.

Since 2000, significant advances in surgery, extracorporeal circulation support, and perioperative management have been achieved; however, the incidence of POCD remains high. Cardiac surgery involves several procedures, including anesthesia, cardiopulmonary bypass, and surgery.^16,17)^ The comparable changes in hemoglobin levels and liver dysfunction in the 2 groups support that cardiac surgery *per se* is an acute stress that results in blood loss and hypoperfusion. The increased white blood cell counts and absence of clinical manifestations of postoperative infection imply that the increased white blood cell counts and transiently elevated serum levels of CRP and IFNγ are attributable to cardiac surgery-mediated acute stress.^18)^ The unchanged levels of CRP and IFNγ in the ulinastatin-treated group suggest a probable protective effect of ulinastatin. In addition, the serum levels of MMP9 and S100β were lower in the ulinastatin-treated group than those in the control group, which is concomitant with improved cognition in the ulinastatin group.

MMP9 is a zinc-dependent protease that is involved in extracellular matrix degradation.^19)^ Increased MMP9 levels are reported in neurological diseases, including ischemic cerebrovascular disease, multiple sclerosis, Alzheimer’s disease, and malignant glioma,^20)^ contributing to the disruption of the blood–brain barrier,^21)^ neuroinflammation, and neuronal damage.^22)^ Sl00β, a neurotrophic factor, is critical for neuropsychological diseases.^23)^ Systemic and local increases in Sl00β correlate with neuronal damage.^23,24)^ Taken together with the comparable serum levels of CRP, IFNγ, TNFα, and IP-10 in the 2 groups, these findings indicate that increased MMP9 and Sl00β levels are key players in POCD in the present study.^25,26)^

TNFα and IP-10, 2 well-studied pro-inflammatory factors, quickly reach their peaks during the operation or early after surgery. Comparable serum levels of TNFα and IP-10 in the present study are probably attributed to their short half-lives.^27)^

The present study had some limitations. Both the MMSE and the Montreal Cognitive Assessment are used in clinical practice. The MMSE can sometimes underestimate impairments due to subjective judgment, while the Montreal assessment is time-consuming, leading to decreased patient compliance. Long-term changes in cognition after surgery are also worthy of attention. However, long-distance follow-up and the COVID-19 epidemic significantly compromised our follow-up evaluation. Therefore, a long-term follow-up is highly recommended in future studies.

## Conclusion

Taken together, medical intervention with ulinastatin improves cognitive function in patients after cardiac surgery by reducing the serum levels of MMP9 and S100β. These findings underscore the need for a better understanding of POCD and its associated medical interventions.

## Declarations

### Data availability

Data sharing is not applicable to this article.

## References

[1] Abrahamov D, Levran O, Naparstek S, et al. Blood-Brain barrier disruption after cardiopulmonary bypass: diagnosis and correlation to cognition. Ann Thorac Surg 2017; 104: 161–9. 10.1016/j.athoracsur.2016.10.043 28193536

[2] Bruno MA, Mufson EJ, Wuu J, et al. Increased matrix metalloproteinase 9 activity in mild cognitive impairment. J Neuropathol Exp Neurol 2009; 68: 1309–18. 10.1097/NEN.0b013e3181c22569 PMC2810197 19915485

[3] Cabral-Pacheco GA, Garza-Veloz I, Castruita-De la Rosa C, et al. The roles of matrix metalloproteinases and their inhibitors in human diseases. Int J Mol Sci 2020; 21: 9739. 10.3390/ijms21249739 PMC7767220 33419373

[4] Casati A, Fanelli G, Pietropaoli P, et al. Monitoring cerebral oxygen saturation in elderly patients undergoing general abdominal surgery: a prospective cohort study. Eur J Anaesthesiol 2007; 24: 59–65. 10.1017/S0265021506001025 16824246

[5] Dow CT, Kidess Z. Proposing Bromo-epi-androsterone (BEA) for perioperative neurocognitive disorders with Interleukin-6 as a druggable target. J Clin Anesth 2025; 101: 111736. 10.1016/j.jclinane.2024.111736 39746239

[6] Farsak B, Gunaydin S, Yorgancioglu C, et al. Elevated levels of s-100beta correlate with neurocognitive outcome after cardiac surgery. J Cardiovasc Surg (Torino) 2003; 44: 31–5. 12627069

[7] Feinstein I, Wilson EN, Swarovski MS, et al. Plasma biomarkers of tau and neurodegeneration during major cardiac and noncardiac surgery. JAMA Neurol 2021; 78: 1407–9. 10.1001/jamaneurol.2021.2823 PMC8453356 34542578

[8] Huang L, Tian W, Chen X, et al. Peripheral neutrophils-derived matrix metallopeptidase-9 induces postoperative cognitive dysfunction in aged mice. Front Aging Neurosci 2022; 14: 683295. 10.3389/fnagi.2022.683295 PMC8902411 35273488

[9] Inouye SK, Westendorp RG, Saczynski JS. Delirium in elderly people. Lancet 2014; 383: 911–22. 10.1016/S0140-6736(13)60688-1 PMC4120864 23992774

[10] Jin Z, Hu J, Ma D. Postoperative delirium: perioperative assessment, risk reduction, and management. Br J Anaesth 2020; 125: 492–504. 10.1016/j.bja.2020.06.063 32798069

[11] Li XF, Zhang XJ, Zhang C, et al. Ulinastatin protects brain against cerebral ischemia/reperfusion injury through inhibiting MMP-9 and alleviating loss of ZO-1 and occludin proteins in mice. Exp Neurol 2018; 302: 68–74. 10.1016/j.expneurol.2017.12.016 29291404

[12] Siqueira GSA, Hagemann PMS, Coelho DS, et al. Can MoCA and MMSE be interchangeable cognitive screening tools? A systematic review. Gerontologist 2019; 59: e743–63. 10.1093/geront/gny126 30517634

[13] Coen RF, Robertson DA, Kenny RA, et al. Strengths and limitations of the MoCA for assessing cognitive functioning: findings from a large representative sample of irish older adults. J Geriatr Psychiatry Neurol 2016; 29: 18–24. 10.1177/0891988715598236 26251108

[14] Agha RA. MG, Rashid R, Kerwan A, et al. TITAN Group. Transparency In The reporting of Artificial INtelligence–the TITAN guideline, 2025.

[15] Lili X, Zhiyong H, Jianjun S. A preliminary study of the effects of ulinastatin on early postoperative cognition function in patients undergoing abdominal surgery. Neurosci Lett 2013; 541: 15–9. 10.1016/j.neulet.2013.02.008 23422155

[16] Liu M, Shen J, Zou F, et al. Effect of ulinastatin on the permeability of the blood-brain barrier on rats with global cerebral ischemia/reperfusion injury as assessed by MRI. Biomed Pharmacother 2017; 85: 412–7. 10.1016/j.biopha.2016.11.044 27916423

[17] Ma Y, Zhao S, Shen S, et al. A novel recombinant slow-release TNF α-derived peptide effectively inhibits tumor growth and angiogenesis. Sci Rep 2015; 5: 13595. 10.1038/srep13595 PMC4559766 26337231

[18] Shen K, Shi Y, Wang X, et al. Cellular components of the blood-brain barrier and their involvement in aging-associated cognitive impairment. Aging Dis 2024; 16: 1513–34. 10.14336/AD.202.0424 PMC12096933 39122454

[19] Siman-Tov T, Bosak N, Sprecher E, et al. Early age-related functional connectivity decline in high-order cognitive networks. Front Aging Neurosci 2017; 8: 330. 10.3389/fnagi.2016.00330 PMC5223363 28119599

[20] Skvarc DR, Berk M, Byrne LK, et al. Post-operative cognitive dysfunction: an exploration of the inflammatory hypothesis and novel therapies. Neurosci Biobehav Rev 2018; 84: 116–33. 10.1016/j.neubiorev.2017.11.011 29180259

[21] Subramaniam B, Shankar P, Shaefi S, et al. Effect of intravenous acetaminophen vs placebo combined with propofol or dexmedetomidine on postoperative delirium among older patients following cardiac surgery: the DEXACET randomized clinical trial. JAMA 2019; 321: 686–96. 10.1001/jama.2019.0234 PMC6439609 30778597

[22] Sun L, Dong R, Xu X, et al. Activation of cannabinoid receptor type 2 attenuates surgery-induced cognitive impairment in mice through anti-inflammatory activity. J Neuroinflammation 2017; 14: 138. 10.1186/s12974-017-0913-7 PMC5518095 28724382

[23] Taylor J, Parker M, Casey CP, et al. Postoperative delirium and changes in the blood-brain barrier, neuroinflammation, and cerebrospinal fluid lactate: a prospective cohort study. Br J Anaesth 2022; 129: 219–30. 10.1016/j.bja.2022.01.005 PMC9465948 35144802

[24] Thelin EP, Nelson DW, Bellander BM. A review of the clinical utility of serum S100B protein levels in the assessment of traumatic brain injury. Acta Neurochir (Wien) 2017; 159: 209–25. 10.1007/s00701-016-3046-3 PMC5241347 27957604

[25] Thomas A, Guo J, Reyes-Dumeyer D, et al. Inflammatory biomarkers profiles and cognition among older adults. Sci Rep 2025; 15: 2265. 10.1038/s41598-025-86309-z PMC11748720 39824904

[26] Yu H, Li H, Liu X, et al. Levels of serum S100B are associated with cognitive dysfunction in patients with type 2 diabetes. Aging (Albany NY) 2020; 12: 4193–203. 10.18632/aging.102873 PMC7093188 32112645

[27] Zhang M, Zhang YH, Fu HQ, et al. Ulinastatin may significantly improve postoperative cognitive function of elderly patients undergoing spinal surgery by reducing the translocation of lipopolysaccharide and systemic inflammation. Front Pharmacol 2018; 9: 1007. 10.3389/fphar.2018.01007 PMC6189321 30356711

